# When evolution is the solution to pollution: Key principles, and lessons from rapid repeated adaptation of killifish (*Fundulus heteroclitus*) populations

**DOI:** 10.1111/eva.12470

**Published:** 2017-04-26

**Authors:** Andrew Whitehead, Bryan W. Clark, Noah M. Reid, Mark E. Hahn, Diane Nacci

**Affiliations:** ^1^ Department of Environmental Toxicology University of California Davis Davis CA USA; ^2^ Atlantic Ecology Division National Health and Environmental Effects Research Laboratory Office of Research and Development Oak Ridge Institute for Science and Education US Environmental Protection Agency Narragansett RI USA; ^3^ Department of Molecular and Cell Biology University of Connecticut Storrs CT USA; ^4^ Department of Biology Woods Hole Oceanographic Institution Woods Hole MA USA; ^5^ Superfund Research Program Boston University Boston MA USA; ^6^ Atlantic Ecology Division National Health and Environmental Effects Research Laboratory Office of Research and Development US Environmental Protection Agency Narragansett RI USA

**Keywords:** adaptation, contemporary evolution, ecological genetics, ecotoxicology, genomics/proteomics, molecular evolution, natural selection and contemporary evolution, population genetics—empirical

## Abstract

For most species, evolutionary adaptation is not expected to be sufficiently rapid to buffer the effects of human‐mediated environmental changes, including environmental pollution. Here we review how key features of populations, the characteristics of environmental pollution, and the genetic architecture underlying adaptive traits, may interact to shape the likelihood of evolutionary rescue from pollution. Large populations of Atlantic killifish *(Fundulus heteroclitus)* persist in some of the most contaminated estuaries of the United States, and killifish studies have provided some of the first insights into the types of genomic changes that enable rapid evolutionary rescue from complexly degraded environments. We describe how selection by industrial pollutants and other stressors has acted on multiple populations of killifish and posit that extreme nucleotide diversity uniquely positions this species for successful evolutionary adaptation. Mechanistic studies have identified some of the genetic underpinnings of adaptation to a well‐studied class of toxic pollutants; however, multiple genetic regions under selection in wild populations seem to reflect more complex responses to diverse native stressors and/or compensatory responses to primary adaptation. The discovery of these pollution‐adapted killifish populations suggests that the evolutionary influence of anthropogenic stressors as selective agents occurs widely. Yet adaptation to chemical pollution in terrestrial and aquatic vertebrate wildlife may rarely be a successful “solution to pollution” because potentially adaptive phenotypes may be complex and incur fitness costs, and therefore be unlikely to evolve quickly enough, especially in species with small population sizes.

## 1 NATURAL SELECTION AND ADAPTIVE RESPONSES TO RAPIDLY CHANGING ENVIRONMENTS

Adaptation to environmental chemicals has marked the history of life, including adaptation to changing oxygen levels, metals’ bioavailability, plant allelochemicals, and natural neurotoxins (Monosson, 2012). However, in contemporary human‐altered environments, the key challenge for life is the degree and pace of environmental change. Can adaptive evolution keep up with rapid and dramatic alterations to the environment? Darwin promoted the perspective that adaptive evolution unfolds over long periods of time, and this view was maintained as a precept in evolutionary thinking and population genetic theory until relatively recently (Hairston, Ellner, Geber, Yoshida, & Fox, 2005; Messer, Ellner, & Hairston, 2016). Experimental and observational research has now demonstrated that adaptation can unfold over very short timescales; well‐known examples include guppy adaptation to predators (Reznick, Shaw, Rodd, & Shaw, 1997), finch adaptation to food availability (Boag & Grant, 1981; Grant & Grant, 2006), fish adaptation to harvest pressure (Conover & Munch, 2002; Olsen et al., 2004), and pest and pathogen adaptation to pesticides and pharmaceuticals (Palumbi, 2001). Indeed, rapid adaptation to pesticides, which are deliberately designed and deployed for their lethal properties, has been extensively documented (e.g., Whalon, Mota‐Sanchez, & Hollingworth, 2008). In contrast, with a few notable exceptions (Antonovics, Bradshaw, & Turner, 1971; Klerks & Levinton, 1989; Klerks, Xie, & Levinton, 2011; Weis, Heber, Weis, & Vaidya, 1981; Weis & Weis, 1989), relatively little is known of the frequency and likelihood of adaptation to environmental pollution (Whitehead, 2014), which results from unintentional release of industrial by‐products into the environment. The pace of adaptive evolution to changing environments depends on characteristics of the environment (e.g., complexity, speed, and severity of environmental change) and characteristics of the species of interest (e.g., population size, generation time).

In this article, we examine the interaction of population genetic processes, environmental stressors, and species attributes that govern the evolutionary fate of species residing in polluted environments. We focus on rapidly and repeatedly evolved pollution tolerance in populations of Atlantic killifish (*Fundulus heteroclitus*), making comparisons to rapid evolutionary examples from the pesticide adaptation literature, and highlighting emerging details of the genomic bases of these evolutionary responses.

### 1.1 Role of generation time, population size, and genetic variation

An irony of evolutionary toxicology is that those species that we intend to kill with chemicals tend to evolve, and those that we would prefer to not kill with chemicals tend toward decline. This contrast is correlated with population size and generation time. Species that we intend to kill tend to have very large population sizes and short generation times (e.g., insects, weeds, bacteria, viruses), and those species that we care about conserving tend to be larger bodied animals with relatively small population sizes and long generation times (e.g., most vertebrates). Short generation times allow evolutionary change to closely track the pace of environmental change (see, e.g., Bergland, Behrman, O'Brien, Schmidt, Petrov, 2014), thus minimizing the mismatch between physiological tolerance and the degree of environmental change. Large population sizes are important to buffer a species from stochastic extinction following rapid population decline in response to abrupt environmental change (Bell & Gonzalez, 2009). Another crucial factor influencing the rate and probability of adaptation is the amount of preexisting genetic variation in evolving populations (Barrett & Schluter, 2008; Orr & Unckless, 2014). This quantity is positively correlated with population size.

### 1.2 Chemical complexity and dimensionality of the adaptive phenotype

The “complexity” and pace of environmental change are important for determining the likelihood of adaptive evolutionary change. If environmental change is complex, requiring simultaneous evolution of multiple independent traits, then adaptation should be relatively slow. For example, increasing atmospheric CO_2_ is simultaneously changing ocean temperature, acidity, and oxygen availability, thus potentially posing concurrent challenges for thermal physiology, shell secretion, and oxygen transport to marine species. Similarly, urban or industrial effluents containing mixtures of chemical compounds may pose concurrent challenges to multiple physiological systems within an organism, and may require simultaneous evolution of diverse pathways and their components to restore fitness. Furthermore, pollutants are often encountered in otherwise degraded environments. For example, in urban estuaries organisms may be exposed to multiple stressors, such as combinations of altered food availability, acoustic stress, reduced visibility, elevated hypoxia, and altered pathogen challenge. Thus, complex suites of stressors may require highly “dimensional” adaptive phenotypes.

Complexity and severity of environmental change also interact with population size to determine the pace and likelihood of adaptive change. Highly dimensional adaptive phenotypes may require adaptive variation in multiple genes to co‐occur, which is more likely in large populations. Similarly, environmental change severe enough to threaten absolute fitness causes rapid demographic decline; species with initially large populations may be sufficiently buffered from stochastic extinction to provide time for adaptive variants to increase in frequency (Bell & Gonzalez, 2009). Furthermore, the rate of environmental change itself may limit the types of genetic variation that can contribute to adaptation. Indeed, different genetic variants may be favored if environmental change is rapid versus gradual. For instance, the adaptive value of mutations that confer tolerance to extreme environments can depend on the potentiating effects of prior mutations that were adaptive to more moderate environments (Lindsey, Gallie, Taylor, & Kerr, 2013).

In contrast to pollution, antibiotics and pesticides are deliberately designed to act by targeting specific biochemical pathways, so one or a few molecular changes can underpin adaptation, which permits relatively rapid adaptation (ffrench‐Constant, Daborn, & Le Goff, 2004). Two relevant contrasts between pesticides and pollutants are related to their chemical nature and how they are encountered in nature. Pesticides (and antibiotics) are designed to be acutely lethal for deliberately targeted species and are accordingly designed to interact with distinct molecular targets with high specificity. For example, many classes of neurotoxic insecticides are sodium channel blockers or acetylcholinesterase inhibitors, and many herbicides disrupt photosynthesis by binding photosystem proteins. Two types of adaptive changes are commonly observed in target species in response to pesticides: changes in target site sensitivity and changes in genes involved in biotransformation or excretion, leading to reduced concentration of the pesticide at its site of action (Taylor & Feyereisen, 1996). Adaptive alterations of a few target proteins seem to repeatedly underpin evolved pesticide tolerance. Across many insect species, mutations in sodium channels confer DDT and pyrethroid tolerance (Soderlund & Knipple, 2003), mutations in GABA channels confer cyclodiene tolerance (Andreev, Kreitman, Phillips, Beeman, & ffrench‐Constant, 1999; Anthony, Unruh, Ganser, & ffrench‐Constant, 1998; ffrench‐Constant, Steichen, Rocheleau, Aronstein, & Roush, 1993), and mutations in AChE confer organophosphate and carbamate tolerance (Fournier, 2005; Lee, Kim, Kwon, Cha, & Kim, 2015), and in many plants mutations in photosystem II proteins confer tolerance to triazine herbicides (Jasieniuk, BruleBabel, & Morrison, 1996). Other adaptive options may be available for evolved metabolic resistance to pesticides: In dozens of species, mutations in multiple gene families, including cytochrome P450 genes, glutathione S‐transferase genes, and esterase genes, have been associated with evolved tolerance to most common classes of pesticides (e.g., DDT, organophosphates, pyrethroids, neonicotinoids) in many species (Bass & Field, 2011; ffrench‐Constant et al., 2004; Li, Schuler, & Berenbaum, 2007).

In contrast to pesticides, industrial pollutants are not acutely toxic by design and occur in complex mixtures, so the toxicity phenotypes are often diverse and nonspecific. For example, globally ubiquitous industrial pollutants such as dioxin‐like chemicals (DLCs) may disrupt cardiovascular and craniofacial development in vertebrate embryos (King‐Heiden et al., 2012), and in adults may cause immune system, liver, and skin toxicity, reproductive dysfunction, and cancer (Denison, Soshilov, He, DeGroot, & Zhao, 2011). Similarly, some polycyclic aromatic hydrocarbons (PAHs), which are common toxicants in combustion pollutants and crude oil, can cause developmental defects in embryos (Barron, Carls, Heintz, & Rice, 2004; Cherr, Fairbairn, & Whitehead, 2017), and immune system toxicity, oxidative stress, endocrine disruption, and cancer in adults (Hylland, 2006). Deliberately designed poisons tend to have discrete mechanisms of acute biological effect, with distinct molecular targets, so that adaptive phenotypes may be simple. In contrast, many industrial pollutants cause toxicity through diverse mechanisms such that adaptive phenotypes may be complex and therefore require relatively long periods of time to evolve. Pesticides and pollution are also encountered differently in nature. Pesticides are often deployed as single or very few chemicals, and in discrete short‐acting applications. However, pollution is often encountered as complex mixtures of chemicals, release is often continuous, and the most concerning pollutants are persistent and bioaccumulative, and therefore long‐acting. Chemicals with multiple mechanisms of action, when encountered together, make adaptation more unlikely, as the adaptive phenotype becomes highly dimensional. Thus, we propose that, although evolutionary adaptation to pesticides and antibiotics is common, adaptation to chemical pollution in wildlife is less likely in part because potentially adaptive phenotypes may be complex and therefore unlikely to evolve quickly, especially in species with small population sizes.

### 1.3 Genetic architecture of rapid evolution

Population genetic theory has developed over the past century within the paradigm that adaptive evolution unfolds over long periods of time under directional and constant selection pressure (Messer et al., 2016). How might very rapid environmental change, which promotes very rapid phenotypic evolution, cause adaptation to unfold differently on a genetic level compared to adaptation to more gradual change? As summarized in the previous section, much of the pesticides adaptation literature highlights the adaptive importance of few genes of large effect, in contrast to polygenic adaptation. This phenomenon may be related to the severity of environmental change: when selection abruptly favors resistance beyond the phenotypic range of sensitivity within a population, rare mutations of large effect are selected (ffrench‐Constant et al., 2004). The evolutionary rescue literature recognizes a distinction between adaptation that is governed by variation in relative fitness and adaptation that emerges from threats on absolute fitness—that is, changes in the environment that are lethal and therefore exceed the distribution of sensitivity phenotypes within populations (Bell, 2013). This interaction between the intensity of selection and the fitness effects of selected variants has been supported by experimental evolution studies, in which different and far fewer genetic variants are favored following rapid environmental change compared to scenarios of equally severe but more gradual change (Lindsey et al., 2013).

Although adaptation to pesticides or drugs may often include few genes of large effect, that does not mean that the adaptive genotype should be simple. Because many genes affect multiple traits, a genetic change of large effect to support pesticide tolerance may have negative impacts on other traits, thereby incurring fitness costs (e.g., Carriere & Roff, 1995; Gassmann, Carriere, & Tabashnik, 2009). Pleiotropic effects of adaptive mutations can impose limits on the evolutionary process (Antonovics, 1976; Lande, 1983). Therefore, following the primary acquisition of chemical tolerance, additional adaptive evolution that compensates for fitness costs may unfold (Lenski, 1988; Uyenoyama, 1986). Thus, even though mutations in a single gene may confer chemical tolerance (e.g., Riveron et al., 2014), one should be cautious in concluding that the adaptive genotype is this simple, as pleiotropic fitness costs may have promoted additional compensatory adaptations at other loci within the genome. Experimental studies have shown that, following gene loss, compensatory evolution elsewhere in the genome is common and swift (Szamecz et al., 2014). Similarly, compensatory adaptation for the fitness costs of antibiotic resistance can quickly evolve in bacteria (Andersson & Hughes, 2010). In the short term, physiological adjustments could at least partially compensate for the negative pleiotropic consequences of rapid adaptation; for example, the functional impacts of specific gene mutations may be rescued through up‐regulation of paralogs that retain functions that are redundant to the mutated gene (Rossi et al., 2015). Presumably compensatory genetic changes would only be favored by selection if physiological compensation were insufficient. Revealing the complete adaptive genotype, including primary resistance and secondary compensatory adaptations, is now feasible through whole‐genome sequencing of many individuals, such that illuminating the complexity of the genomic landscape that underlies rapid adaptation is within reach.

Genomewide studies of rapid adaptation may be particularly useful for revealing the subset of locally adaptive genotypes that are compensatory, because when adaptive change is rapid and abrupt, fitness costs could be particularly strong (Lande, 1983; Xie & Klerks, 2004) and selection pressure for compensatory adaptations may be amplified. Experimental studies have shown that the extent of compensatory adaptation positively scales with fitness impacts of particular mutations (Szamecz et al., 2014). Population genomics scans help reveal both directly adaptive and compensatory loci, and quantitative trait locus (QTL) mapping may further clarify those directly associated with specific adaptive phenotypes. Further clarifying compensatory loci is more challenging; systems biology may help to predict the unintended side effects of chemicals or adaptive evolution through modeling of how perturbation of specific molecular receptors and pathways may influence the function of interacting proteins and pathways (Xie, Li, Xie, & Bourne, 2009). One could then generate hypotheses about the genes likely to be subject to compensatory adaptive fine‐tuning. Genomewide studies of adaptation to synthetic chemicals also offer opportunity to understand the complexity of adaptive phenotypes at a level of detail that is rarely achieved for other types of adaptive phenotypes. This is because there exists a rich literature on the distinct molecular mechanisms of biological activity for many of these chemicals, such that we may start to dissect primary adaptive change (chemical tolerance) from secondary (compensatory) adaptive change.

### 1.4 Evolutionary toxicological systems as models

The study of evolutionary toxicology is multidisciplinary in nature. It requires, at a minimum, the integration of expertise from evolution, population biology, molecular biology, and biochemistry. Each of these fields is experiencing rapid technological and/or theoretical development, so evolutionary toxicology studied in an integrative framework is well‐placed to yield insight not only into applied problems in environmental science and human health, but also basic conceptual problems in biology. For example, a fundamental question in biology is how the phenotype maps to the genotype, but this is currently poorly understood. In evolutionary toxicology study systems, where response to chemical exposure (phenotype) determines organismal fitness, natural selection may act on genetic variation to alter that map and mitigate the effects of environmental perturbation. Those fitness‐restoring variants are of basic interest in evolutionary biology because their origin, the distribution of their fitness effects, and how the structure of the genotype–phenotype map shapes those factors are not yet well‐known. They are also of basic interest in molecular biology, the goal of which is to understand developmental and regulatory processes at a molecular level. Systems in which evolution has occurred in response to toxic exposure could be thought of as forward genetic screens in which the pool of mutations has been enriched for those that are functionally and environmentally significant—a potentially very different set than might be uncovered through genomewide association in populations not subject to natural selection, or through mutagenic approaches. A useful feature of evolutionary toxicology systems is that they tend to be replicated. Many environmentally damaging human activities (oil spills, mining, agriculture, etc.) occur repeatedly in discrete locations, applying similar selection pressures across populations and species of organisms in ways that strengthen conclusions that can be drawn from studying them.

## 2 INTRODUCTION TO THE KILLIFISH STORY: RAPID ADAPTATION TO EXTREME ENVIRONMENTAL POLLUTION

### 2.1 Killifish natural history

Atlantic killifish (*F. heteroclitus*) present a compelling study system for revealing the mechanisms that may contribute to rapidly evolved adaptation to environmental pollution. This is because they are abundant in coastal marsh ecosystems (Valiela, Wright, Teal, & Volkmann, 1977) including polluted urban estuaries, are genetically variable (Duvernell, Lindmeier, Faust, & Whitehead, 2008), and are nonmigratory with small home ranges (Lotrich, 1975; Teo & Able, 2003), so locally specialized phenotypes may evolve quickly. They are a model system in evolutionary physiology (Burnett et al., 2007); populations have evolved variation in thermal physiology along Atlantic coastal temperature gradients (Powers et al., 1993) and variation in osmoregulatory physiology along salinity gradients (Whitehead, Roach, Zhang, & Galvez, 2011). They have also rapidly evolved tolerance to environmental toxicants that pollute urban estuaries (Nacci, Champlin, & Jayaraman, 2010; Weis, 2002; Weis & Weis, 1989). These tolerance phenotypes can be heritable and dramatic. Because tolerance to some chemicals has evolved independently multiple times (Nacci et al., 2010), powerful comparative methods may be brought to bear. Pollution tolerance has also evolved in populations of their sister species *Fundulus grandis* (Oziolor, Bigorgne, Aguilar, Usenko, & Matson, 2014). Furthermore, much is known about the molecular mechanisms of action for the major chemical pollutants to which they have adapted, thereby providing a foundation for inferring the function of genes and pathways showing signatures of adaptive divergence. *Fundulus heteroclitus* has a reference genome sequence (Reid et al., in press), and much is known about their transcriptome‐wide response to relevant chemical toxicants, including differences between tolerant and sensitive populations (Bozinovic & Oleksiak, 2010; Bozinovic, Sit, Di Giulio, Wills, & Oleksiak, 2013; Fisher & Oleksiak, 2007; Oleksiak, 2008; Oleksiak et al., 2011; Whitehead, Pilcher, Champlin, & Nacci, 2012; Whitehead, Triant, Champlin, & Nacci, 2010).

### 2.2 Four distinct environments drive adaptive evolution

To document the diversity of habitat in which killifish reside, fish and sediment have been sampled at dozens of locations that range over 5 orders of magnitude in sediment polychlorinated biphenyl (PCB) concentrations, where PCBs are used as a proxy for urban and industrialized global contamination (Nacci et al., 2010). Even at the most contaminated of these sites, where pollutants exceed lethal levels and killifish are not expected to persist (e.g., Munns et al., 1997), they appear to thrive; their unexpected occurrence has resulted in intense study to better understand “how can they survive here”?

Studies have focused on four sites within the killifish range, each polluted with unique patterns of contamination including mixtures of persistent, toxic, and bioaccumulative industrial pollutants, such as metals and aromatic and halogenated aromatic hydrocarbons (AHs and HAHs, respectively) (Nacci et al., 2010; Van Veld & Nacci, 2008; Whitehead et al., 2012; Wirgin & Waldman, 1998). Each of these sites is located near an EPA‐designated urban/industrial estuarine Superfund site. From north to south, these sites include New Bedford Harbor, Massachusetts, heavily contaminated with PCBs; Bridgeport, Connecticut, heavily contaminated with urban pollutants including PCBs and AHs; Newark, New Jersey, heavily contaminated with dioxins; and the Atlantic Wood site in the Elizabeth River, Virginia, contaminated with PAHs from creosote. Some AHs are referred to collectively as DLCs to reflect their mechanistic similarities to the highly toxic family of HAHs known as “dioxins” (described later in this review). Some PAHs share similar molecular responses and toxicity phenotypes with DLCs, although their metabolism and persistence can differ markedly from DLCs. Within this broad categorizations of DLCs and PAHS are also important compound‐specific aspects of mechanisms of action that influence chemical‐specific toxicity and potential adaptive mechanisms.

#### 2.2.1 New Bedford Harbor (MA)

Although typical of many urban estuaries of the northeast coast of the USA in terms of nutrient over‐enrichment, habitat loss, and other characteristics of anthropogenic disturbance (Nelson et al., 1996), NBH sediment and biota contain extraordinarily high concentrations of PCBs (Lake, McKinney, Lake, Osterman, & Heltshe, 1995; Nelson et al., 1996; Pruell et al., 1990). Industrial wastes were discharged directly into the harbor from the 1940s to the 1970s, producing contamination of sufficient magnitude to warrant listing on the US EPA's National Priorities List as a Superfund site (Bergen, Nelson, Mackay, Dickerson, & Jayaraman, 2005; Nelson et al., 1996). Sediment PCB levels near discharge were measured as high as 100,000 μg/g dry weight (Weaver, 1984), and at other areas of the Superfund site have been measured as 2,100 μg/g dry weight in NBH (total PCBs) (Pruell et al., 1990), or >10,000 times greater than the sediment guideline value for total PCBs that has been correlated with probable adverse biological effects (180 ng/g dry weight) (Long, Macdonald, Smith, & Calder, 1995). Estimated from chemical analyses of dated sediment cores, PCB concentrations in sediments at the Superfund site have been at toxic levels for decades (Latimer et al., 2003; McMillan, Bagley, Jackson, & Nacci, 2006). Although PCB discharge ceased in 1976 (www.epa.gov/nbh), biota sampled from NBH more than 20–30 years later continue to bioaccumulate PCBs (Fritsch et al., 2015; Grans et al., 2015; Lake et al., 1995). For example, the mean concentration for total PCBs in livers of killifish collected in 1996 from the upper harbor Superfund site was 324 μg/g dry weight (Nacci, Champlin, Coiro, McKinney, & Jayaraman, 2002). In comparison, the mean concentration of total PCBs in livers of killifish from West Island (WI), a reference site outside NBH, was 2.4 μg/g dry weight (Nacci et al., 2002). Yet even in the region of highest chemical contamination, there are acres of marshy shoreline opposite the industrialized side of the NBH that provide ample habitat for killifish, which are abundant throughout NBH. The larger ecological effects of NBH chemical contamination per se have not been well documented; however, reduced species richness and diversity was reported in this Superfund estuary (Mitchell & Oviatt, 1996).

#### 2.2.2 Bridgeport (CT)

Bridgeport is a densely populated, major urban center along the Northeast corridor, where intense pollution warranted an investigation of the potential ecological effects of ocean disposal of contaminated material from Black Rock Harbor (BRH). These sediments were highly polluted with PAHs, PCBs, and many other toxic contaminants (Rogerson, 1988) that contributed to adverse biological effects in laboratory tests using estuarine species (e.g., Gardner, Yevich, Harshbarger, & Malcolm, 1991). Killifish collected from an accessible shoreline site nearby to BRH were shown to be DLC‐tolerant and phenotypically similar to other tolerant killifish populations (Nacci et al., 2010; Whitehead et al., 2010, 2012). Like other urban estuaries, habitat quality is poor, and surficial sediment contamination at the collection site was shown to be moderately high in proxy contamination (PCBs) (Nacci et al., 2010), but likely contaminated with many other pollutants associated with BRH, a nearby landfill, sewage treatment plant, and other industries.

#### 2.2.3 Newark

The entire New York New Jersey Harbor area is notorious for its intense sediment pollution associated with the most densely populated, major urban center along the Northeast corridor. These sediments have been characterized as highly polluted in dioxins, PAHs, PCBs, and many other toxic contaminants (e.g., Iannuzzi, Armstrong, Thelen, Ludwig, & Firstenberg, 2005). Early studies described tolerance characteristics in killifish from Newark (Prince & Cooper, 1995a, 1995b). Killifish collected adjacent to the former “Newark Yacht Club” were shown to be DLC‐tolerant and phenotypically similar to other tolerant killifish populations (Elskus, Monosson, McElroy, Stegeman, & Woltering, 1999; Nacci et al., 2010; Whitehead et al., 2012). Like other urban estuaries, habitat quality is poor, and surficial sediment contamination was moderately high in proxy contamination (PCBs) (Nacci et al., 2010), but known to be contaminated with many other pollutants associated with all manner of industrial and human‐use activities.

#### 2.2.4 Elizabeth River

The Elizabeth River is a heavily industrialized subestuary of the lower Chesapeake Bay, surrounded by the cities of Chesapeake, Norfolk, Portsmouth, and Virginia Beach, Virginia. The river has been a prominent military, commercial, and industrial hub throughout US history, dating back to the colonial era. As with other urbanized estuaries, these activities have contributed to substantial degradation, including habitat loss, contamination with bacteria and nutrients from municipal effluents, and pollution by heavy metals, and PCBs from various industries. However, unlike the other sites discussed here, the dominant contaminants in the Elizabeth River are PAHs from coal tar creosote. Creosote, developed for wood treatment in the 1830s, is used to protect telephone poles, railroad ties, and marine pilings from marine wood borers and microbial decay (Nicholas, 1982). Coal tar creosote is a distillation of the remainder from burning coal, yielding a complex mixture of aromatic hydrocarbons. Unlike petrogenic mixtures such as crude oil, creosote consists almost wholly of aromatic hydrocarbons, including significant amounts of the 3–5 ring compounds to which much of the toxicity is often attributed. Creosote also includes nitrogen‐, oxygen‐, and sulfur‐containing heterocyclic compounds.

Although it is unclear how many creosote plants existed on the Elizabeth River, at least three major creosote treatment and storage facilities operated from at least the early 1900s—Eppinger and Russell, Republic Creosoting, and Atlantic Wood Industries (Merrill & Wade, 1985). The contamination is spatially heterogeneous throughout the estuary, but the highest measured concentrations of total PAHs (740–1730 μg/g dry weight) by far have been measured near the former creosote facilities (Walker, Dickhut, & Chisholm‐Brause, 2004). The Atlantic Wood Industries site was added to the National Priorities List as a Superfund Site in 1990, and its resident killifish population has been the subject of much study (reviewed in Di Giulio & Clark, 2015). Habitat quality varies widely in the Elizabeth River, and the Atlantic Wood Industries site is in a small inlet with relatively little marsh habitat, isolated by a deep water shipping channel, shipyards, and developed shoreline.

## 3 NATURE OF PARALLEL POLLUTION ADAPTATION IN KILLIFISH

**Figure 1:**
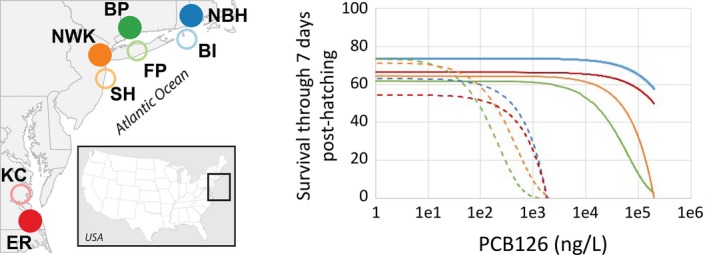
Population variation in killifish larval survival when challenged with increasing exposure concentrations of PCB126. Modeled responses from repeated laboratory tests show that populations from polluted sites (solid curves) exhibit tolerance to pollutants at concentrations hundreds to thousands of times normally lethal levels (sensitive populations, dashed curves). Populations are indicated by colors as shown in the map, which shows locations of tolerant populations (solid circles: New Bedford Harbor [NBH], Bridgeport [BP], Newark [NWK], and Elizabeth River [ER] from north to south) and sensitive reference populations (open circles: Block Island [BI], Flax Pond [FP], Sandy Hook [SH], and Kings Creek [KC], from north to south). PCB, polychlorinated biphenyl

Killifish populations resident to the four highly contaminated sites highlighted above, where sediment PCB contamination greatly exceeds probable adverse effects levels (Long et al., 1995), have been shown to be highly tolerant of the DLC, PCB126, with lethal effect (LC20) concentrations 576‐ to more than 8,333‐times higher than for killifish from relatively clean sites (Figure 1) (Nacci et al., 2010). The sensitivity to PCB126 of these four most tolerant populations as measured in the progeny of wild‐collected fish (the F1 generation) was ranked as Newark ~ Bridgeport > New Bedford > Elizabeth River. Furthermore, for three of these populations, tolerance in F1 and F2 generations appeared relatively similar in magnitude. This dramatically reduced sensitivity (tolerance) was most striking for the Elizabeth River population, where no adverse effects were observed at the highest tested PCB126 concentration for the F1 or F2 generation. Similar responses across generations were also evident for the New Bedford and Newark killifish populations; however, there appeared to be a degradation of tolerance between the F1:F2 generations for the Bridgeport killifish (Nacci et al., 2010).

In addition to their tolerance to PCB126, fish from these sites exhibit tolerance to a variety of other DLCs, including chlorinated dioxins, chlorinated dibenzofurans, and PAHs (Bello, Franks, Stegeman, & Hahn, 2001; Nacci et al., 1999). A systematic comparison among sites for the degree of sensitivity to these other compounds has not been made, so the extent to which the relative tolerance to different types of DLCs varies among sites is not known.

Field conditions that are toxic to killifish occur at sites far less contaminated than Superfund sites (Nacci et al., 2002, 2010). Among many sites representing a range of sediment PCB contamination spanning over four orders of magnitude, the level of PCB pollution was inversely correlated with sensitivity of resident killifish, based on laboratory bioassays. This evidence of widespread adaptive variation in DLC sensitivity (Nacci et al., 1999, 2010) supports the conclusion that genetic adaptation to DLCs occurs at sites that are not extraordinarily contaminated, that is, at concentrations of sediment PCBs equal or greater than concentrations correlated with probable ecological effects (i.e., 180 ng/g) (Long et al., 1995). Extrapolating to other geographic areas and ecosystems, this suggests that the evolutionary influence of contaminants and other anthropogenic stressors as selective agents occurs on a geographic scale much larger than expected.

### 3.1 Evidence for adaptation from physiology and developmental biology

Early investigations into the effects of contaminants in polluted sites occupied by killifish focused on their bioaccumulation (e.g., Mothershead, Hale, & Greaves, 1991; Rappe et al., 1991) and acute toxicity and carcinogenicity in adult fish (e.g., Hargis, Roberts, & Zwerner, 1984; Hargis, Zwerner, Thoney, Kelly, & Warinner, 1989; Johnson et al., 1993). However, it was soon discovered that embryonic, larval, and adult Atlantic killifish from polluted sites were refractory to the induction of the cytochrome P450 1A (CYP1A) response that is the hallmark biomarker of exposure to many DLCs and PAHs that were in such abundance at these sites (Bello et al., 2001; Elskus et al., 1999; Nacci et al., 1999; Powell, Bright, Bello, & Hahn, 2000; Prince & Cooper, 1995a; Van Veld & Westbrook, 1995).

**Figure 2:**
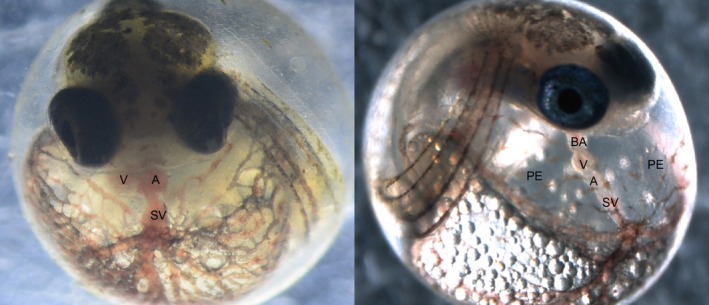
Comparison of a normally developed embryo (left) and a PCB‐affected embryo (right) at 10 days postfertilization. Exposure to dioxin‐like chemicals, including some PCBs, causes a suite of developmental effects, including reduced embryo size, swim bladder malformation, alteration of jaw cartilage, vascular hemorrhaging, pericardial and yolk‐sac edema, and malformation of the heart. On the right, a severely affected fish exhibits a heart that resembles a tube, unable to generate substantial blood flow, accompanied by massive pericardial edema. On the left, a normally developed embryo has a compact heart, with a ventricle and atrium aligned side by side under the jaw. Fish that have evolved tolerance show very limited signs of these developmental defects following chemical exposure, even at doses thousands of times higher than those that cause these effects in fish from reference (clean) sites. These developmental impacts are ultimately lethal. V, ventricle; A, atrium; PE, pericardial edema; BA, bulbus arteriosus; SV, sinus venosus; PCB, polychlorinated biphenyl

Embryos from populations recalcitrant to CYP1A induction also exhibited remarkable resistance to the developmental toxicity caused by the DLCs and PAHs and contaminated sediments to which they were experimentally exposed (Meyer & Di Giulio, 2002; Nacci et al., 1999; Ownby et al., 2002; Prince & Cooper, 1995a). In fish embryos, DLCs normally produce a suite of developmental defects that came to be known as blue‐sac disease after their appearance in lake trout exposed to dioxin in the US Great Lakes (Cook et al., 2003; Tillitt, Cook, Giesy, Heideman, & Peterson, 2008). This toxicity is characterized by reduced embryo size, defects in swim bladder formation, alteration of jaw cartilage, vascular hemorrhaging in the yolk sac, brain, and tail, pericardial and yolk‐sac edema, and malformation of the heart (King‐Heiden et al., 2012). In normally developing embryos, the heart consists of the sinus venosus, which consolidates blood flow from the network of vessels surrounding the yolk sac, a thin‐walled atrium, a thick‐walled ventricle responsible for the generation of pumping pressure, and the bulbus arteriosus, which controls blood flow out of the heart. Following exposure to some DLCs and PAHs in early development, severely affected fish exhibit hearts that resemble a single thin tube unable to generate substantial blood flow, usually accompanied by significant pericardial edema (Figure 2). Even at lower concentrations or with less potent DLCs, the ultrastructure of the heart is still significantly altered, with reduction in the size of the chambers, alteration of the alignment of the chambers, and reshaping of the sinus venosus and bulbus arteriosus. These developmental effects are absent or dramatically reduced in embryos from tolerant populations.

### 3.2 Evidence for adaptation from transcriptomics

The killifish research community was an early adopter of functional genomics approaches to study mechanisms underlying adaptive divergence between populations (Oleksiak, Churchill, & Crawford, 2002; Oleksiak, Kolell, & Crawford, 2001). The first studies comparing pollution‐adapted to sensitive killifish populations used curated microarrays of metabolic genes to probe brain and liver gene expression in laboratory‐acclimated adult animals, and found extensive divergence in constitutive expression, particular in xenobiotic transformation genes (Fisher & Oleksiak, 2007; Oleksiak, 2008). However, few of these metabolic genes were differentially expressed in common among multiple tolerant populations. Application of the next generation of genomewide microarray tools revealed striking differences in pollutant responses between tolerant and sensitive populations, particularly during early life, and striking parallelism among tolerant populations. Almost no differences in constitutive gene expression were apparent between tolerant and sensitive populations during development in clean, “common‐garden” conditions (Bozinovic & Oleksiak, 2010). However, upon exposure to model toxicants, including PCBs and PAHs, large differences in inducible expression clearly distinguished tolerant fish from fish from a nearby sensitive reference population (Bozinovic et al., 2013; Oleksiak et al., 2011; Whitehead et al., 2010). These results show that the functional divergence in gene expression between populations is dependent on exposure to toxicants—an example of population‐by‐environment interaction.

**Figure 3:**
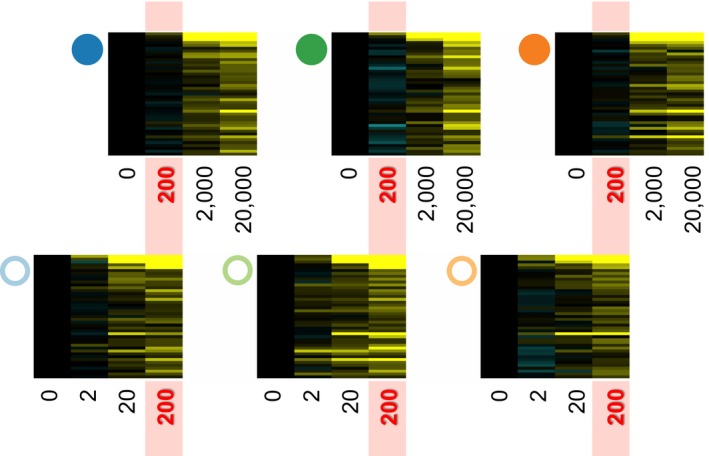
Differences in the transcriptional response to PCB126 exposure among northern tolerant and sensitive killifish populations. Populations indicated by colored circles (filled or open) as in Figure 1. Heatmaps show genes that differ in their response to PCB exposure. Columns are treatments, including control and exposure concentrations of PCB126 (ng/L), rows are genes, and the color of cells indicates expression relative to control (black) where yellow and blue represent up‐ and down‐regulation, respectively. Populations from reference sites show a common up‐regulation of a suite of genes that is enriched for the transcriptional targets of an activated AHR signaling pathway. Tolerant populations from polluted sites show little transcriptional response to the same exposures that cause large gene expression changes in sensitive reference fish. However, the transcriptional response of tolerant fish becomes similar to that of sensitive fish after the dose is increased two to three orders of magnitude. This demonstrates that AHR activation is profoundly desensitized, but not completely disabled, in killifish populations that have evolved pollution tolerance. PCB, polychlorinated biphenyl; AHR, aryl hydrocarbon receptor

When multiple populations were compared side by side in their response to PCB exposure, extensive convergence in patterns of inducible gene expression was detected among tolerant populations compared to their reference populations (Figure 3), suggesting repeated evolution of a similar adaptive phenotype (Reid et al., 2016; Whitehead et al., 2012). Data from all of these studies consistently show that adaptation is associated with global desensitization of the aryl hydrocarbon receptor (AHR) signaling pathway. This pathway is normally activated by binding HAHs and PAHs to the AHR. AHR activation during embryonic development leads to altered gene expression, malformations, and decreased survivorship (Billiard, Meyer, Wassenberg, Hodson, & Di Giulio, 2008; King‐Heiden et al., 2012). Experimental knockout or knockdown of AHR function protects against HAH‐ and PAH‐induced toxicity (Billiard, Timme‐Laragy, Wassenberg, Cockman, & Di Giulio, 2006; Clark, Matson, Jung, & Di Giulio, 2010; Fernandez‐Salguero, Hilbert, Rudikoff, Ward, & Gonzalez, 1996; Goodale et al., 2012; Incardona, Day, Collier, & Scholz, 2006; Incardona et al., 2005; Jönsson, Jenny, Woodin, Hahn, & Stegeman, 2007; Prasch et al., 2003); thus, evolved desensitization of this signaling pathway is plausibly causative for adaptive pollutant tolerance in killifish.

## 4 AHR PATHWAY

### 4.1 Roles of AHR in gene regulation and toxicity

The reduced sensitivity of killifish inhabiting polluted sites to both the characteristic embryotoxic effects of DLCs and some PAHs, and the induction of gene expression signatures that characterize exposures to those chemicals (e.g., *CYP1A* and other genes), implicated the AHR pathway as likely being involved in the adaptive mechanism. The AHR is a ligand‐activated transcription factor that was initially identified because of its role in mediating dioxin (TCDD; 2,3,7,8‐tetrachlorodibenzo‐p‐dioxin) toxicity and in regulating the induction of xenobiotic‐metabolizing enzymes in response to TCDD and PAHs in mice (Burbach, Poland, & Bradfield, 1992; Poland, Glover, & Kende, 1976). The AHR is a member of the basic helix‐loop‐helix, Per‐Arnt‐Sim (bHLH‐PAS) family of genes involved in developmental signaling and environmental sensing (Gu, Hogenesch, & Bradfield, 2000; McIntosh, Hogenesch, & Bradfield, 2010); other well‐known bHLH‐PAS genes include the hypoxia‐responsive transcription factors *HIF‐1α* and *HIF‐2α* and the circadian clock proteins *CLOCK, PER,* and *BMAL*.

The canonical mechanism of AHR signaling is initiated by high‐affinity binding of ligands such as TCDD, PCB126, or PAHs, which leads to nuclear localization of the AHR complex, dissociation of chaperone proteins hsp90 and AHR‐interacting protein (AIP), and association with the nuclear bHLH‐PAS protein AHR nuclear translocator (ARNT). The ligand‐bound AHR‐ARNT complex interacts with specific enhancer sequences (AHR response elements or AHREs) on target genes to regulate the expression of those genes. The transient activation of AHR and subsequent induction of xenobiotic‐metabolizing enzymes is considered an adaptive response, facilitating the biotransformation and excretion of toxic endogenous and exogenous compounds. However, sustained activation of AHR, such as occurs by exposure to poorly metabolizable DLCs or constant exposure to labile AHR agonists, leads to toxicity through sustained misregulation of genes involved in cellular homeostasis. The AHR is thus an integral component of the mechanism by which DLCs and some PAHs cause toxicity, and loss of AHR function protects against these effects (Fernandez‐Salguero et al., 1996; Goodale et al., 2012; Mimura et al., 1997; Prasch et al., 2003). Although the AHR is now known to participate in a variety of additional, noncanonical interactions and pathways (Jackson, Joshi, & Elferink, 2015), the primary mechanism of toxicity appears to involve canonical interactions requiring nuclear localization, dimerization with ARNT, and binding to AHREs (Bunger et al., 2003, 2008; Nukaya, Walisser, Moran, Kennedy, & Bradfield, 2010; Walisser, Bunger, Glover, Harstad, & Bradfield, 2004).

As important as the AHR appears to be in controlling adaptive and toxic responses to DLC and PAH exposure, those are not its only functions. Results from a variety of laboratories over the past decade have revealed that the AHR has key roles in the development of cardiovascular, reproductive, and neural systems as well as in hematopoiesis and regulation of immune responses (Benedict, Lin, Loeffler, Peterson, & Flaws, 2000; Esser & Rannug, 2015; Gasiewicz, Singh, & Bennett, 2014; Kimura, Ding, & Tohyama, 2016; Lahvis et al., 2005; Quintana & Sherr, 2013; Singh et al., 2014; Stockinger, Di Meglio, Gialitakis, & Duarte, 2014). Some of these functions may involve natural or endogenous ligands such as metabolites generated by the microbiome, tryptophan metabolites, or lipid‐derived molecules (Bessede et al., 2014; Hubbard, Murray, & Perdew, 2015; McMillan & Bradfield, 2007; Moura‐Alves et al., 2014). The exact relationship between the AHR‐dependent toxicity of DLCs and PAHs and these physiological roles of AHR is not yet clear. However, the existence of these physiological roles suggests that a reduction in AHR signaling may be accompanied by physiological fitness costs.

### 4.2 AHR variation among vertebrates and role in differential sensitivity

Although much of our understanding of the AHR pathway has come from studies in mammalian systems, the fundamental features of AHR function appear to be broadly conserved in vertebrate animals (Hahn, 1998a). Thus, AHR regulates adaptive and toxic responses to DLC and some PAH exposures in fish as in mammals, and loss of AHR function ameliorates these effects (Billiard et al., 2006; Goodale et al., 2012; Jönsson et al., 2007; Prasch et al., 2003). Despite the fundamental similarities, however, there are also differences in the AHR pathway among vertebrates—including fish—that may be important in their adaptive evolutionary response to pollution. A major difference is in the number of AHR genes. In contrast to the single AHR gene present in most mammals, fish (and most other vertebrates) have multiple AHR genes (Hahn, Karchner, & Merson, 2017; Hahn et al., 2006). Fish typically have four AHR genes, products of a tandem gene duplication (*AHR1* and *AHR2*) and paralogs of each of these (designated “a” and “b”), the result of a whole‐genome duplication at the base of the teleost lineage, after its divergence from the lineage leading to tetrapods (Hahn et al., 2006, 2017). The relative roles of each AHR are not yet fully understood, but the AHR2 paralogs appear to mediate many of the toxic and adaptive responses to DLCs and some PAHs (Antkiewicz, Peterson, & Heideman, 2006; Billiard et al., 2006; Clark et al., 2010; Dong, Teraoka, Tsujimoto, Stegeman, & Hiraga, 2004; Goodale et al., 2012; Incardona et al., 2006; Jönsson et al., 2007; Lanham, Prasch, Weina, Peterson, & Heideman, 2011; Prasch et al., 2003; Waits & Nebert, 2011). For some chemicals, however, responses may be mediated by AHR1 (Goodale et al., 2012; Incardona et al., 2006).

In addition to being dependent on the presence of a functioning AHR, the toxicity of DLCs and some PAHs is also influenced by the properties of AHRs and by the nature of ligand–AHR interactions. Among HAHs, ligand affinity is a good predictor of toxicity; compounds that bind with greatest affinity are the most potent at altering gene expression and causing toxic effects (Safe, 1994). Differences in AHR properties, including affinity for ligand binding, explain differences in sensitivity to DLCs in several vertebrate systems, including strains of rodents (Moffat, Roblin, Harper, Okey, & Pohjanvirta, 2007; Poland, Palen, & Glover, 1994), species of birds (Farmahin et al., 2013; Karchner, Franks, Kennedy, & Hahn, 2006), and populations of some fish (Atlantic tomcod) (Wirgin et al., 2011). (At the extreme end of this spectrum are invertebrates, which have AHR proteins that lack the ability to bind DLCs [Powell‐Coffman, Bradfield, & Wood, 1998; Butler, Kelley, Powell, Hahn, & Van Beneden, 2001] and also are largely insensitive to DLC toxicity [Hahn, 1998a].) Ligand affinity is not the only factor that controls sensitivity, however. There are several other “control points” in the AHR signaling cascade that can influence the sensitivity to or magnitude of response (Beischlag, Luis Morales, Hollingshead, & Perdew, 2008; Hahn, 1998b). Similarly, the AHR is not the only factor that influences sensitivity to effects of DLCs; the expression and function of other members of the AHR pathway, including ARNT (Nukaya, Walisser, et al., 2010; Tomita, Sinal, Yim, & Gonzalez, 2000; Walisser et al., 2004) and AIP (Nukaya, Lin, et al., 2010), also are important. In addition, the expression and inducibility of biotransformation enzymes can influence sensitivity, especially for labile AHR ligands such as PAHs (Billiard et al., 2008; Brown, Clark, Garner, & Di Giulio, 2015). Thus, there are multiple possible paths to reduced sensitivity to AHR ligands.

## 5 GENETIC BASIS OF POLLUTION TOLERANCE IN KILLIFISH

### 5.1 Evidence from candidate gene and SNP approaches

Theoretical considerations (Hoffmann & Willi, 2008; Macnair, 1991; Woods & Hoffman, 2000) suggest that adaptation of killifish to DLCs would involve major gene effects rather than polygenic adaptation (discussed in Reitzel et al.; 2014). In light of the key role of AHR in controlling sensitivity in a variety of vertebrates, an initial focus was on AHR itself as a candidate resistance gene, a hypothesis complicated by the identification of multiple killifish AHRs (Karchner, Powell, & Hahn, 1999). Several polymorphic variants of both *AHR1* and *AHR2* were identified, and both loci (now known as *AHR1a* and *AHR2a*) showed signals of selection in the NBH population (Hahn, Karchner, Franks, & Merson, 2004; Reitzel et al., 2014). However, functional analysis of multiple variants in vitro did not reveal any differences in ligand‐binding properties or ability to activate gene expression (Hahn et al., 2004; Karchner, Franks, Middleton, & Hahn, 2017).

Surveys of allelic variation within and between multiple tolerant and sensitive populations were conducted for a suite of genes known to be functionally associated with the AHR signaling pathway (Proestou, Flight, Champlin, & Nacci, 2014; Reitzel et al., 2014). These were predicted to be targets of selection based on the altered function of the AHR pathway in tolerant compared to sensitive populations. Genes included components of the AHR pathway, nuclear receptors known to “cross talk” with the AHR pathway (i.e., estrogen receptors, retinoic acid receptors, hypoxia‐inducible factors), cytochrome P450s, genes involved in cardiac development, cathepsins, and genes having oxidoreductase activity. Patterns of allelic differentiation consistent with local pollution adaptation were detected for genes *AHR1*,* AHR2*,* cathepsin Z*,* CYP1A*, and the NADH dehydrogenase subunits (Proestou et al., 2014; Reitzel et al., 2014). Thus, genotyping results for tolerant populations suggest that tolerance correlates with variation among a suite of loci including genes known to be related to the AHR pathway, whose poor inducibility by DLCs and PAHs is a hallmark of evolved killifish tolerance. An additional marker that stood out was another cytochrome P450, *CYP3A30*, whose activity may correlate with responses to non‐dioxin‐like PCBs (Chang et al., 2013; Grans et al., 2015), which predominate at PCB‐contaminated sites.

### 5.2 Evidence from QTL mapping

Direct tests for association of genetic variation with tolerance phenotypes (cardiovascular abnormalities in developing killifish exposed to PCB126) were conducted to characterize the genetic basis of tolerance in the NBH population using a QTL mapping approach. For this purpose, F1 progeny were produced by cross‐breeding mature killifish from NBH and Block Island, RI, one of the most sensitive populations ever tested (Nacci et al., 2010), and when mature these F1 fish were crossed to produce F2 embryos. These F2 embryos were exposed to a single differentiating concentration of PCB126 and phenotyped during development, demonstrating their representation of the range of sensitivity between the two originating populations (e.g., Whitehead et al., 2010). F2 embryos were genotyped using a dense panel of markers including targeted SNPs (Proestou et al., 2014) and microsatellite markers (Waits et al., 2016). The statistical linkage between phenotype and genetic markers was accomplished using a QTL mapping approach (Nacci, Proestou, Champlin, Martinson, & Waits, 2016). Mapping was conducted before the *F. heteroclitus* reference genome was available, so QTL markers were aligned to other fish genomes to reveal the identity of nearby genes.

**Figure 4:**
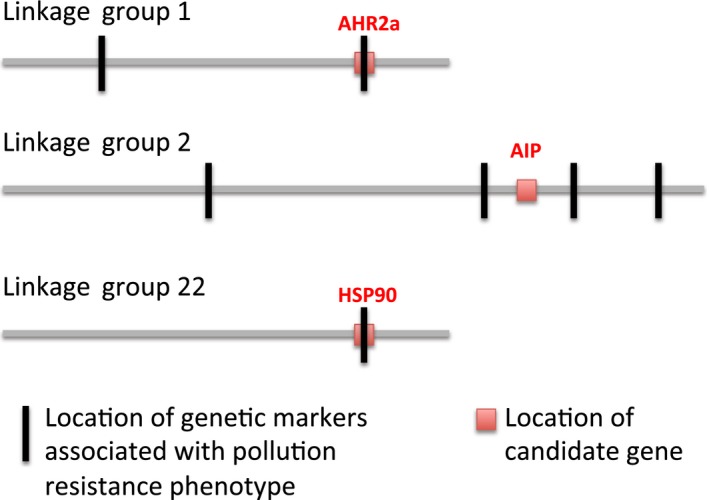
Genetic markers identified as quantitative trait loci (black) associated with tolerance to dioxin‐like compounds (DLCs) in New Bedford Harbor (MA) killifish, and some candidate genes (red) known for their involvement in the aryl hydrocarbon receptor (AHR) signal transduction pathway through which DLCs act (Nacci, Proestou, et al., 2016)

Seven main QTL intervals associated with NBH tolerance were identified on three linkage groups or putative chromosomes, as well as one additive and three epistatic interactions (Nacci, Proestou, et al., 2016). These QTLs accounted for 69% of the variance associated with tolerance to DLCs. It was unsurprising that *AHR2a* emerged as a QTL, given its role in DLC toxicity, and given signals of selection detected previously in AHR genes (Hahn et al., 2004; Proestou et al., 2014; Reitzel et al., 2014). However, the *AHR2a* QTL had relatively low explanatory power for tolerance in killifish (17%). Variants near other AHR pathway genes also appeared correlated with the tolerance phenotype. For example, the most significant QTL identified, explaining up to 46% of the phenotypic variance within families analyzed (Nacci, Proestou, et al., 2016), was nearby to the gene encoding *aryl hydrocarbon receptor interacting protein* (*AIP*, also known as *HBV x‐associated protein 2*,* XAP2*, and *AH receptor‐activated 9*,* ARA9*; Trivellin & Korbonits, 2011) (Figure 4). While the roles of AIP are not precisely known and may vary by species and life stage, it is known to modulate the function of AHR signaling (Petrulis, Kusnadi, Ramadoss, Hollingshead, & Perdew, 2003), and influences DLC toxicity (Nukaya, Lin, et al., 2010). Another genetic marker near *AIP* interacted significantly as an additive QTL with *HSP90*. This potential for a combination of variants (*AHR2*,* AIP*,* HSP90*) affecting the AHR‐ligand binding complex suggests both a mechanism for tolerance to DLCs, and an explanation for why variation in AHR alone only partially accounts for tolerance in killifish. Thus, the QTL approach identified genetic regions associated with DLC tolerance that included some genes known for their involvement in the AHR signal transduction pathway through which DLCs act, as well as other markers whose roles in DLC tolerance are not yet known.

### 5.3 Evidence from population genomics

A population genomics analysis was designed and conducted to discover regions of the tolerant genome exhibiting signatures of recent natural selection (Reid et al., 2016). Whole genomes were re‐sequenced at low coverage for 48 individuals from each of eight populations, including from four pollution‐tolerant populations and for each of those from a nearby reference (sensitive) population (the populations included in Figure 1). Four pairs of tolerant‐sensitive populations were each scanned for signatures of natural selection, including for genomic regions with unusually high allele frequency differentiation between the population pair and for regions with reduced nucleotide diversity in the tolerant member of the population pair. For each tolerant‐sensitive population pair, a second sensitive population was then added to the comparison to confirm signatures of selection associated with the tolerant member of the contrast. Within each population pair, genomic regions showing selection signatures were then ranked according to the strength of the selection signal, where the rank score was a combination of the size of the selected region and the extremity of differentiation and reduction in nucleotide diversity. Many dozens of selection signature regions were discovered for each tolerant population, yet only a few of these regions were shared among all tolerant populations. However, the top‐ranking outliers for each population tended to be among those few that were shared across all tolerant populations. Therefore, although the degree of genomic convergence among tolerant populations was small, the genes with likely the greatest putative fitness benefits in polluted sites have convergently evolved in those populations.

**Figure 5:**
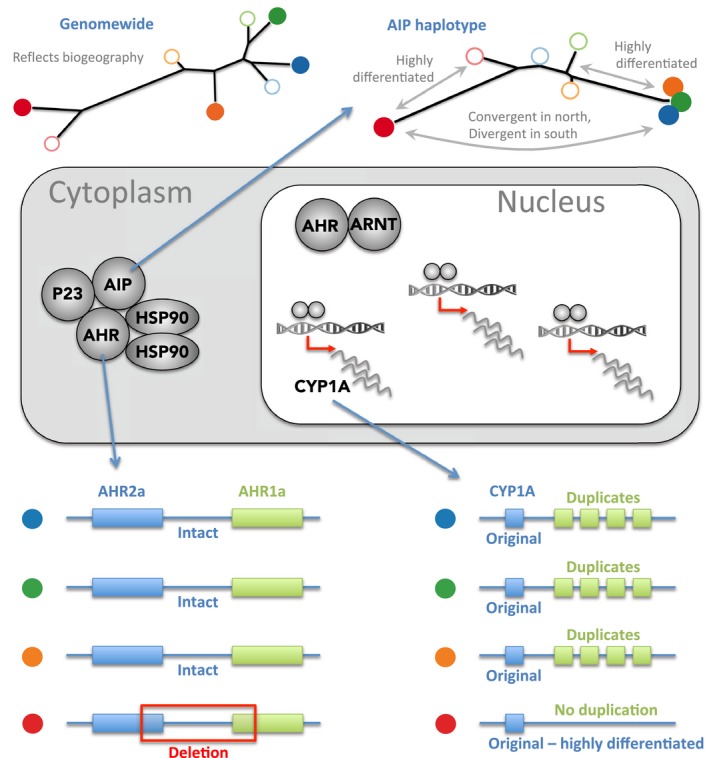
Genomic regions showing strongest signatures of selection between tolerant‐sensitive pairs of populations are shared among tolerant populations, and include genes in the AHR signaling pathway, indicating convergent adaptation at the pathway and gene levels. However, different molecular variants are implicated in different populations. Populations indicated by colored circles (filled or open) as in Figure 1. Phylogenetic tree at the top left was estimated from genomewide bi‐allelic SNP frequencies, and shows the expected biogeographic pattern of relatedness among populations: geographically nearby populations cluster together, and northern populations are distinct from southern. The center panel illustrates the key proteins associated with the AHR signaling pathway. Among the strongest signals of selection are genes *AHR*,*AIP*, and *CYP1A*. For *AIP*, a common haplotype was favored by selection in northern populations, but a distinct haplotype diverged in the ER population (phylogenetic tree at top right). The genomic region around *AHR2a* and *AHR1a* is highly divergent between tolerant and sensitive populations, but only in the ER (red circle) population is this selection signal associated with a deletion that has swept to high frequency (bottom left panel). *CYP1A* falls in a region of strong selection for all tolerant populations, but only in the northern populations is this signal associated with up to eightfold duplication of the *CYP* *1A* locus (bottom right panel). *AHR, aryl hydrocarbon receptor; CYP1A, cytochrome P450 1A; AIP, AHR‐interacting protein*

Within the top‐ranked and shared outlier regions are the genes *AHR1a/2a*,* AIP*, and *CYP1A* (Figure 5). *AHR1a* and *AHR2a* are adjacent, separated by only 15 kb. While the two genes fall together in the center of a region apparently under selection in all four sampled tolerant populations, molecular variants found in each are different. In three populations, alleles with deletions that impact both genes are present. In one of these populations, a deletion spanning the gap between *AHR1a* and *AHR2a* is found at ~80% frequency; intriguingly, a chimeric transcript is expressed that is composed of fragments of the two genes. The impact of these deletion variants on the functioning of AHR signaling and their contribution to the tolerance phenotype remains to be discovered.

*AIP* falls in the center of one of the highest ranking outlier regions in all four populations. In this case, however, the data indicate only two major haplotype variants: one shared by the three northern tolerant populations and a second found only in the southernmost tolerant population. The first haplotype is close to fixation in two populations (BP, NWK), and at ~82% frequency in the third (NBH). The second haplotype exists only in the ER population and is close to fixation. Both haplotypes have a core region of about 100 kb unbroken by recombination, which contains many variants showing high frequency differences between tolerant and sensitive populations. For these two haplotypes, however, we detected no variants (nucleotide or structural) that are both shared among them and that distinguish them from all sensitive reference populations. This suggests that the causal variants in each haplotype are different, although because there are some small gaps in the reference assembly in this region, we cannot exclude the possibility that we did not detect the causal variant.

*CYP1A* falls in another very highly ranked outlier region in all four populations. In this region, we again see divergence between variants likely under selection in the northern tolerant populations and the southern. In this case, the re‐sequencing data indicate a series of duplications of the region containing *CYP1A* in the three northern populations, but not in the southern population. Individuals from tolerant populations in the north have up to eight copies of *CYP1A* per diploid genotype (mean = 4) while individuals from the southern tolerant population and all sensitive populations have an average of 2 (maximum 4 in a single individual in a northern sensitive population); thus, there is no evidence of duplication in the southern tolerant population. In northern populations, there is evidence for three separate duplication events with different genomic spans. Short read data and the local signature of selection indicate that at least some of the extra copies are inserted in tandem.

A number of other AHR signaling pathway genes are implicated by these data, including genes that regulate AHR signaling and genes that are transcriptional targets of AHR activation. For example, *AHR1b/2b* are within outlier regions in two tolerant populations. Five additional genes (*HSP90, ARNT, CYP1C1/1C2, GRFP, and GST‐theta*) are in outlier regions in only the southern tolerant population (Reid et al., 2016).

Experimental studies suggest that when selection is very strong, only a few molecular variants offer viable adaptive options (Lindsey et al., 2013), such that shared adaptive solutions are more likely to evolve than unique ones. In other words, convergent evolution is more likely under abrupt or severe environmental change. Similarly, mathematical modeling predicts that as the strength of selection and population size increases, the likelihood of convergent evolution increases (MacPherson & Nuismer, 2017). When adaptive variation with large phenotypic effects preexist as standing genetic variation, this also contributes to the likelihood of convergent evolution. For multiple loci to evolve in parallel, very strong selection and very large populations are particularly important. Nucleotide diversity in Atlantic killifish is among the highest of any vertebrate species (Reid et al., 2016, in press), and adaptive variation appears to have existed within populations before the rise of chemical pollution. This high diversity likely enabled the unusual adaptive success of killifish in urban estuaries, especially because selection was very strong because sediments contain concentrations of contaminants well above the lethal limits for killifish from clean sites. Only a small subset of genes showed convergent patterns of adaptive evolution across multiple tolerant populations, yet these genes also harbored the strongest signatures of selection in each tolerant‐sensitive pair (Reid et al., 2016). This supports the prediction that under very strong selection, especially in species with very large population sizes, convergent evolution is more likely. Furthermore, these data indicate that adaptive solutions to ubiquitous and persistent organic pollutants may be constrained through adaptive modification of just a few key molecular pathways.

Many more genes showed signatures of selection in wild killifish populations than were associated with phenotypic tolerance in QTL mapping studies. Signatures of selection in wild populations presumably reflect a complex adaptive phenotype, including adaptation to chemicals with different modes of action (e.g., non‐DLC PCBs, endocrine disruptors, metals), adaptation to other stressors in these radically altered habitats (e.g., hypoxia, altered community), compensatory adaptations to the negative pleiotropic consequences of primary tolerance‐conferring adaptations, and perhaps adaptations that confer fitness advantage at specific life stages. In contrast, the QTL mapping approach is designed to identify population differences related to specific traits of experimental interest, for example, tolerance to a specific chemical structure like PCB126. Together, these complementary approaches offer great potential for revealing the genetic architecture that underpins adaptation.

### 5.4 More than AHR: a complex evolved response to a complex suite of stressors

AHR pathway genes are consistently differentially responsive in all tolerant populations compared to their sensitive counterparts, and AHR pathway genes harbor among the strongest signatures of selection in all tolerant populations (Reid et al., 2016). AHR pathway genes are also genetically linked with the tolerance phenotype in developing embryos of killifish and other fish species (Nacci, Proestou, et al., 2016; Waits & Nebert, 2011). The evolutionary modification of this pathway is therefore a crucial and convergent component of the adaptive phenotype in polluted habitats. However, AHR pathway genes contribute only a small fraction of the suite of genes showing signatures of selection in polluted populations, and most of the additional genes showing signatures of selection are unique to different tolerant populations (Reid et al., 2016). What unites all of the four polluted sites where tolerant killifish have been characterized (to date) is contamination with DLCs (dioxins, PCBs) or PAHs. Because toxicity of dioxins and some PCBs and PAHs is either wholly or partially mediated by AHR signaling (e.g., Clark et al., 2010; Prasch et al., 2003), this suggests constraint—at least at a physiological level—on the adaptive options available for natural selection. However, polluted sites are also contaminated with chemicals that do not exert toxicity through the AHR pathway, such as noncoplanar PCBs (Gooch, Elskus, Kloepper‐Sams, Hahn, & Stegeman, 1989; Hestermann, Stegeman, & Hahn, 2000; Walker & Peterson, 1991) and low molecular weight PAHs (Incardona et al., 2005); many of these occur at higher concentrations than the DLCs and AHR agonist PAHs (Clark, Cooper, Stapleton, & Di Giulio, 2013; Nacci et al., 2010). One may predict that some components of the adaptive genotype are associated with tolerance to these other classes of chemicals that contaminate polluted sites, and that site‐specific toxicants may drive population‐specific selection signatures. The population genomics data are consistent with these predictions.

Multidrug resistance (MDR) transport proteins are efflux pumps that often serve as the first line of cellular defense upon exposure to toxic chemicals by preventing their intracellular accumulation (Bard, 2000). A homolog to human *MRP1* (multidrug resistance‐associated protein 1, which confers resistance to anticancer drugs in humans) is within the top‐ranking selection signature region in NBH fish. Some environmental toxicants may be transported from cells by these proteins, whereas others can sensitize organisms to other xenobiotics by blocking MDR function and preventing their export. Contaminants that are common in urban estuaries, such as personal care products, pesticides, heavy metals, and PCBs, may interact with MDR proteins and disrupt their function (Anselmo, van den Berg, Rietjens, & Murk, 2012; Bain & LeBlanc, 1996; Leslie, Deeley, & Cole, 2001; Nicklisch et al., 2016; Shuilleabhain et al., 2005; Smital et al., 2004; Tampal, Robertson, Srinivasan, & Ludewig, 2003). Although many chemicals pollute the NBH site, including heavy metals, PAHs, and pesticides (Deshpande, Dockum, Cleary, Farrington, & Wieczorek, 2013; Pruell et al., 1990), acute toxicity of NBH sediment seems mostly due to PCBs (Ho, McKinney, Kuhn, Pelletier, & Burgess, 1997). Plausible adaptive roles of evolved MDR1 in NBH fish (Bard, Bello, Hahn, & Stegeman, 2002) may include elevated cellular export of conjugated PCBs (Lohitnavy et al., 2008), or reduced PCB‐induced inhibition of the protein's efflux function (Nicklisch et al., 2016; Tampal et al., 2003).

Embryo‐larval cardiotoxicity of some low molecular weight PAHs is independent of AHR signaling (Brown et al., 2015; Incardona, Linbo, & Scholz, 2011; Incardona et al., 2005, 2006, 2014), and may be mediated through disrupted regulation of intracellular potassium and calcium (Brette et al., 2014). Within top‐ranked selection signature regions specific to the ER population, we find genes for two proteins that make up the conductance pore of the voltage‐gated potassium channel (*KCNB2*,* KCNC3*) in cardiomyocytes and *RYR3* that regulates intracellular calcium. What distinguishes the ER site from other polluted sites is extreme contamination with complex mixtures of PAHs (Walker et al., 2004). Adaptive modification of these genes is plausibly associated with resistance to the cardiotoxic effects of PAHs.

Many pollutants in urban estuaries may act as endocrine disruptors (Oberdorster & Cheek, 2001) through interference with estrogen receptor signaling (Shanle & Xu, 2011) and altered estrogen signaling has been observed in tolerant populations of killifish (Bugel, Bonventre, White, Tanguay, & Cooper, 2014; Bugel, White, & Cooper, 2011; Greytak & Callard, 2007; Greytak, Tarrant, Nacci, Hahn, & Callard, 2010). Estrogen receptor 2b is within a selection signature region in the BP population. Pollutants abundant at the BP site, such as PCBs, may have interfered with estrogen receptor (Bonefeld‐Jorgensen, Andersen, Rasmussen, & Vinggaard, 2001; Korach, Sarver, Chae, Mclachlan, & Mckinney, 1988) thereby altering endocrine signaling (Soto et al., 1995) in BP fish, prompting adaptation of the *ESR2b* gene.

Other alterations of urban estuaries from human activities may have had direct impacts on killifish fitness and acted as selective agents. For example, increased turbidity, abundance of invasive species, altered flow regimes, hypoxia, and altered pathogen communities may co‐occur with chemical pollution in urban estuaries, and act as selective agents. Alternatively, killifish may compensate for these other habitat changes through physiological or behavioral flexibility. The molecular targets of adaptive evolution of traits relevant to these stressors are less obvious than for chemical toxicants for which much is known of mechanisms of action. Future experiments, including exploring the dozens of additional selection signature genes in killifish populations adapted to life in urban estuaries, offer opportunity to further illuminate the genetic basis supporting fitness in these radically altered habitats.

## 6 ECOLOGICAL CONSIDERATIONS

### 6.1 Cross‐tolerance to diverse chemicals

In pest populations that become resistant to pesticides, cross‐resistance to new pesticides can present significant challenges for pest control. As discussed previously, this is at least in part because while there are hundreds of different pesticides in use, they have a relatively small set of related molecular targets (Casida, 2009). For example, insects can develop cross‐resistance to DDT and pyrethroids because both of those classes of chemicals commonly inhibit voltage‐gated sodium channels in the central nervous system (e.g., Burton et al., 2011). Shared features of the chemical tolerance phenotype of the multiple tolerant killifish populations are analogous. The contamination in New Bedford Harbor and Bridgeport is dominated by PCBs, in Newark by dioxins, and in the Elizabeth River by PAHs, yet all four populations are resistant to DLCs and PAHs, regardless of their degree of prior exposure to the specific compounds tested. While PAHs do not bind the AHR as strongly as many dioxins or PCBs, there is good evidence that mixtures combining specific PAHs that are AHR agonists with other PAHs that are CYP inhibitors increases their dioxin‐like effects (Wassenberg & Di Giulio, 2004). Given their similar evolved tolerance for chemicals with a shared mechanism of action, it is perhaps expected that killifish from each of the four tolerant populations should be at least partially cross‐resistant to the contaminated environment at any of the other sites.

Evolved tolerance to chemicals including pesticides may also be achieved through enhanced biotransformation (ffrench‐Constant, 2013; Nkya, Akhouayri, Kisinza, & David, 2013), which can confer cross‐tolerance to other chemicals that are metabolized by similar biotransformation pathways. There is evidence that evolved alteration of biotransformation pathways may protect DLC‐tolerant killifish from other toxic chemicals too. For example, down‐regulation of the AHR pathway and a corresponding diminished CYP1 response would be expected to be beneficial for exposure to the neurotoxic insecticide chlorpyrifos, which is bioactivated by CYP1, and in fact Elizabeth River larvae are resistant to chlorpyrifos (Clark & Di Giulio, 2012). In contrast, down‐regulation of the AHR pathway would be expected to sensitize fish to compounds detoxified by CYP1 enzymes, such as neurotoxic insecticides like the pyrethroid permethrin or the carbamate carbaryl, but Elizabeth River larvae were also resistant to these chemicals, as are DLC‐tolerant populations of *F. grandis* (Oziolor, Dubansky, Burggren, & Matson, 2016). These results suggest that evolved mechanisms other than CYP1‐mediated metabolism contribute to cross‐protection from some neurotoxic pesticides.

### 6.2 Costs of evolved tolerance

Ecological and evolutionary theories suggest that adaptation to one form of environmental stress may increase susceptibility to other stressors, also referred to as fitness costs or fitness trade‐offs (Futuyma, 1998). This idea is based on the assumption that adaptation to a new environment involves alterations in a previous, optimal phenotype that had been shaped by the various selection pressures of the ancestral environment (Coustau, Chevillon, & ffrench‐Constant, 2000). Alterations present in the new phenotype should be deleterious to survival in the ancestral environment. Within the context of adaptation to chemical contaminants, traits that are adaptive for specific chemical contaminants may present disadvantages under alternate conditions, referred to as “between environment‐trade‐offs” (Shirley & Sibly, 1999). Indeed, fitness trade‐offs are often detected in pesticide‐ and antibiotic‐resistant populations (Andersson & Hughes, 2010; Uyenoyama, 1986). However, costs to antibiotic resistance are not always observed (Andersson & Hughes, 2010), and given sufficient time in a new environment, the costs of adaptation may eventually be resolved by additional compensatory adaptation (Lenski, 1988; Uyenoyama, 1986).

Reports of fitness costs in tolerant killifish are mixed. For example, reduced fitness manifested as changes in the behavior of individuals and population age‐structure in Newark killifish (Weis, 2002). In contrast, NBH killifish are reproductively prolific when raised in a clean environment (Nacci et al., 2002); they show healthy responses to pathogen challenges (Nacci et al., 2009), have high condition indices (Nacci, Jayaraman, & Specker, 2001), and nearly normal levels of stored vitamin A (Nacci et al., 2001). Vitamin A is considered a sensitive indicator of DLC toxicity in many vertebrate species (Fletcher, Hanberg, & Håkansson, 2001). Complementary studies suggest that demographic compensation (e.g., increased reproductive effort) and/or migration (i.e., from less contaminated populations) do not play important roles in supporting a persistent population in NBH (Nacci, Walters, Gleason, & Munns, 2008; Nacci et al., 2002). However, Meyer and Di Giulio (Meyer & Di Giulio, 2003) reported poor condition (microbial infection, poor growth, emaciation, and increased mortality) in Atlantic Wood killifish maintained in clean water relative to reference fish. In addition, Atlantic Wood offspring were more susceptible to mortality associated with infectious disease (Frederick, Van Veld, & Rice, 2007), to toxicity associated with photoactivation of PAHs, and to acute hypoxia (Meyer & Di Giulio, 2003), although they were less susceptible to the model pro‐oxidant *tert*‐butyl hydroperoxide than reference fish (Meyer, Smith, Winston, & Di Giulio, 2003). Altered estrogen signaling has been observed in DLC‐tolerant killifish from both Newark and NBH (Bugel et al., 2011, 2014; Greytak & Callard, 2007; Greytak et al., 2010). NBH killifish are more sensitive to the pro‐oxidant chemical *tert*‐butylhydroquinone as compared to fish from a reference site (Harbeitner, Hahn, & Timme‐Laragy, 2013); however, the opposite effect was seen in ER fish, which were more tolerant of the oxidant *tert*‐butylhydroperoxide than reference fish (Meyer et al., 2003). Thus, the presence of fitness costs associated with adaptation may depend on specific features of the environment in addition to those of the adaptive phenotype.

### 6.3 Compensation for costs of evolved tolerance

The existence of nontoxicological roles of AHR in immune, reproductive, neural, and cardiovascular function and the cross talk that exists between AHR and other signaling pathways such as those involving estrogens, oxidants, and hypoxia (Puga, Ma, & Marlowe, 2009) is consistent with the idea that adaptive suppression of the AHR pathway in fish at polluted sites may have physiological costs. Several population genomic outliers identified in one or more tolerant populations, including those in regions harboring estrogen receptor *(ESR2b)* and hypoxia‐inducible factor *(HIF‐2α)* genes, may reflect possible compensatory changes (Reid et al., 2016).

Another likely set of compensatory changes occurs at the *CYP1A* locus, which encodes a key xenobiotic‐metabolizing enzyme. *CYP1A* was identified previously as a locus under selection in tolerant populations (Proestou et al., 2014; Williams & Oleksiak, 2011). In the population genomic study, *CYP1A* duplications were found in three of the four tolerant populations; in the three northern tolerant populations, individuals have up to eight *CYP1A* copies (Reid et al., 2016). Even in the absence of exogenous chemical exposure the AHR may help to maintain basal CYP1A expression; loss of AHR signaling in mice reduces this expression (Schmidt, Su, Reddy, Simon, & Bradfield, 1996). Thus, *CYP1A* duplications might have been favored in tolerant fish with suppressed AHR signaling to maintain constitutive levels of CYP1A needed for physiological functions. Although the situation is more complicated in fish, where AHR paralogs may help to maintain CYP1A expression if the function of one AHR is lost (e.g., Goodale et al., 2012), the reduction in AHR signaling in tolerant killifish through changes to both individual AHRs and their binding partners suggests a more pervasive impairment of AHR function. In contrast to the northern populations, there was no evidence of duplication in the southern tolerant population, although this region showed a strong signature of selection. The fact that the primary contaminants at the southern site are PAHs, which can be bioactivated by CYP1A, may have mitigated the benefits of duplication at this locus. However, both chemical and morpholino blockade of CYP1A actually increase the dioxin‐like toxicity of PAHs, suggesting significant benefit to CYP activity for that population (Matson et al., 2008; Wassenberg & Di Giulio, 2004). Thus, the different chemical pollutants acting as selective agents may require finely tuned compensatory changes (Reid et al., 2016).

### 6.4 How will populations change following environmental cleanup?

An important question is whether pollution sensitivity will be restored in populations following cleanup of the contamination, as has been observed in some cases (Levinton et al., 2003). If evolved tolerance in killifish closely tracks the degree of habitat pollution, as suggested by empirical models (Nacci et al., 2010), then one might predict that the pace of population reversion to a sensitive phenotype could be used to track the success of habitat cleanup activities. Reversion to sensitivity could emerge as a consequence of either natural selection disfavoring the tolerance phenotype due to the fitness costs in a clean environment, or as a consequence of migration from nearby historically un‐altered habitats diluting out the resident tolerant genotype. However, phenotypes of lower tolerance may not be strongly favored following habitat recovery—or at least not as strongly favored as tolerance phenotypes were following habitat contamination. In fact, one of the contaminated sites in the Elizabeth River (Eppinger and Russell) has undergone significant cleanup and restoration beginning in the mid‐2000s, but fish from this site are still strongly tolerant of DLCs (Clark et al., 2013). Furthermore, because connectivity between polluted habitats and relatively clean habitats is typically limited, migration of fish from historically uncontaminated sites into recently remediated sites could be very slow (Nacci et al., 2008). For these reasons, reversion of locally adapted tolerant populations from polluted sites to sensitive phenotypes may not closely track the pace of habitat recovery following cleanup.

### 6.5 Implications of killifish tolerance for trophic transfer of pollutants

The Superfund sites that serve as habitat for tolerant killifish were so designated because their levels of contamination in sediment and water are sufficient to increase ecological and human health risks. But the risks may extend beyond the site boundaries. Distribution of Superfund site contamination outside of the designated area can occur not only through physical transport but also through the consumption of contaminated prey by piscivorous, migratory birds, and fish. Thus, ecological costs associated with environments where tolerant killifish flourish include trophic transfer, which can result in bioaccumulation of contaminants in top predators, fish‐eating birds and other wildlife that use the ecosystem as a feeding ground. For example, Nacci, Hahn, et al. (2016) conducted a longitudinal study of eggs from two tern species collected from Buzzards Bay breeding colonies to assess historical and contemporary effects of PCBs on these species whose breeding colonies may have been (or may continue to be) influenced by NBH estuarine Superfund site contamination. Buzzards Bay common terns *Sterna hirundo* and the federally listed roseate terns (RTs) *Sterna dougallii* are exposed to PCBs primarily by ingestion of fish within their feeding range (Nisbet, 2002), which includes NBH. High concentrations of total PCBs displaying the characteristic signature of PCBs from NBH sediment were observed in Buzzards Bay tern eggs collected in the 1970s, while NBH PCB contamination was probably at its maximum. However, like the NBH sediment, PCBs in Buzzards Bay tern eggs collected in subsequent decades, that is, after PCB discharge into NBH ceased, declined, and lost its characteristic pattern of congeners. This and other studies (e.g., Deshpande et al., 2013) suggest that killifish resident to highly contaminated sites extend the range of “evolutionary effects” of pollutants beyond the Superfund site boundaries and potentially beyond resident species.

## 7 CONCLUSIONS

Can adaptive evolution keep up with rapid and dramatic alterations to the environment? Consistent with evolutionary theory, critical factors influencing the rate and probability of adaptation include the amount of preexisting genetic variation in evolving populations, which is positively correlated with population size. Furthermore, complexity and severity of environmental change interact with population size to determine the pace and likelihood of adaptive change. Large population sizes and extreme genetic variation both likely have contributed to the rapid evolutionary adaptation observed in multiple populations of Atlantic killifish. These examples of rapidly evolved pollution tolerance provide opportunity to explore shared and unique genetic mechanisms that support persistence in diverse, extreme, and rapidly changing habitats.

One class of toxic pollutants emerged as a strong agent of selection across killifish populations, and mechanistic knowledge of this class of toxic pollutants has aided interpretation of some genetic alterations observed. However unlike systems driven by single selective agents (like pesticides), killifish population genomic studies have also shown the complex pattern of genes under selection in the wild. While extrapolation of these observations confirms that adaptation in a rapidly changing, human‐altered habitats will not be simple or typically fast enough to provide rescue for most species, the killifish system will continue to serve as a valuable model to predict and test how the interaction of ecology and evolution contribute to the ability of species to respond to rapidly changing environmental conditions.
